# Supplementary material for: Modelling Skylarks (Alauda arvensis) to Predict Impacts of Changes in Land Management and Policy: Development and Testing of an Agent-Based Model
Source: PLoS One. 2013 Jun 6;8(6):e65803. doi: 10.1371/journal.pone.0065803 (PMC3675089; doi:10.1371/journal.pone.0065803)
Supplement: Supporting Information S4 — The skylark ODdox as a zipped archive. (ZIP) [file pone.0065803.s004.zip › Skylark_ODdox/classsk_territory__struct.html]

ALMaSS Skylark ODdox: skTerritory\_struct Class Reference


ALMaSS Skylark ODdox
 2.0


- Main Page
- Related Pages
- Classes
- Files

- Class List
- Class Index
- Class Hierarchy
- Class Members

Public Attributes

skTerritory\_struct Class Reference

`#include <skylarks_all.h>`

List of all members.

|  |  |
| --- | --- |
| Public Attributes | |
| double | nqual |
| int | ref |
| int | size |
| bool | validnest |
| int | x |
| int | y |

---

## Member Data Documentation

double skTerritory\_struct::nqual

Referenced by Skylark\_Male::OnMateLeaving(), Skylark\_Male::ReEvaluateTerritory(), and Skylark\_Female::st\_Finding\_Territory().

int skTerritory\_struct::ref

Referenced by Skylark\_Male::ConstructAHabitatTable(), Skylark\_Male::EstablishingATerritory(), Skylark\_Female::EstablishTerritory(), Skylark\_Male::OnEvicted(), Skylark\_Female::OnMateDying(), Skylark\_Female::OnMateHomeless(), Skylark\_Male::OnMateLeaving(), Skylark\_Male::OnReHouse(), Skylark\_Male::ReEvaluateTerritory(), Skylark\_Adult::Skylark\_Adult(), Skylark\_Male::st\_AttractingAMate(), Skylark\_Female::st\_Dying(), Skylark\_Male::st\_Dying(), Skylark\_Male::st\_FindingTerritory(), Skylark\_Male::st\_FollowingMate(), Skylark\_Female::st\_GivingUpTerritory(), and Skylark\_Female::st\_StoppingBreeding().

int skTerritory\_struct::size

Referenced by Skylark\_Male::EstablishingATerritory(), Skylark\_Female::EstablishTerritory(), Skylark\_Male::OnReHouse(), and Skylark\_Male::st\_FindingTerritory().

bool skTerritory\_struct::validnest

Referenced by Skylark\_Male::OnReHouse(), Skylark\_Male::ReEvaluateTerritory(), and Skylark\_Male::st\_FindingTerritory().

int skTerritory\_struct::x

Referenced by Skylark\_Male::EstablishingATerritory(), Skylark\_Female::EstablishTerritory(), Skylark\_Male::OnEvicted(), Skylark\_Male::OnReHouse(), Skylark\_Male::st\_Arriving(), and Skylark\_Male::st\_FindingTerritory().

int skTerritory\_struct::y

Referenced by Skylark\_Male::EstablishingATerritory(), Skylark\_Female::EstablishTerritory(), Skylark\_Male::OnEvicted(), Skylark\_Male::OnReHouse(), Skylark\_Male::st\_Arriving(), and Skylark\_Male::st\_FindingTerritory().

---

The documentation for this class was generated from the following file:

- skylarks\_all.h


- skTerritory\_struct
- Generated on Thu Jan 10 2013 13:15:37 for ALMaSS Skylark ODdox by
   1.8.1.1
